# Bacterial Infection-Mimicking Three-Dimensional Phagocytosis and Chemotaxis in Electrospun Poly(ε-caprolactone) Nanofibrous Membrane

**DOI:** 10.3390/membranes11080569

**Published:** 2021-07-28

**Authors:** Seung-Jun Lee, Perry Ayn Mayson A Maza, Gyu-Min Sun, Petr Slama, In-Jeong Lee, Jong-Young Kwak

**Affiliations:** 1 Department of Pharmacology, School of Medicine, Ajou University, Worldcup-Ro 164, Suwon 16499, Korea; blacksky@naver.com (S.-J.L.); perryayn@gmail.com (P.A.M.A.M.); rbals3922@ajou.ac.kr (G.-M.S.); 2 Department of Biomedical Sciences, The Graduate School, Ajou University, Suwon 16499, Korea; 3 Department of Animal Morphology, Physiology and Genetics, Faculty of AgriSciences, Mendel University in Brno, Zemedelska 1, 613 00 Brno, Czech Republic; petr.slama@mendelu.cz; 4 3D Immune System Imaging Core Center, Ajou University, Suwon 16499, Korea; injeong@ajou.ac.kr

**Keywords:** nanofiber, poly(caprolactone), 3D culture, neutrophil, dendritic cell, inflammation, *Staphylococcus aureus*

## Abstract

In this study, we developed a three-dimensional (3D) in vitro infection model to investigate the crosstalk between phagocytes and microbes in inflammation using a nanofibrous membrane (NM). Poly(ε-caprolactone) (PCL)-NMs (PCL-NMs) were generated via electrospinning of PCL in chloroform. *Staphylococcus aureus* and phagocytes were able to adhere to the nanofibers and phagocytes engulfed *S. aureus* in the PCL-NM. The migration of phagocytes to *S. aureus* was evaluated in a two-layer co-culture system using PCL-NM. Neutrophils, macrophages and dendritic cells (DCs) cultured in the upper PCL-NM layer migrated to the lower PCL-NM layer containing bacteria. DCs migrated to neutrophils that cultured with bacteria and then engulfed neutrophils in two-layer system. In addition, phagocytes in the upper PCL-NM layer migrated to bacteria-infected MLE-12 lung epithelial cells in the lower PCL-NM layer. *S. aureus*-infected MLE-12 cells stimulated the secretion of tumor necrosis factor-α and IL-1α in 3D culture conditions, but not in 2D culture conditions. Therefore, the PCL-NM-based 3D culture system with phagocytes and bacteria mimics the inflammatory response to microbes in vivo and is applicable to the biomimetic study of various microbe infections.

## 1. Introduction

The recognition of pathogenic microorganisms is the first step of host defense. Components derived from bacteria, such as N-formylmethionine-leucyl-phenylalanine (fMLP), trigger chemotaxis and activation of phagocytes such as neutrophils and macrophages in infectious tissues [1]. Early-recruited neutrophils phagocytose the bacteria and subsequent chemoattractant signals from the activated and necrotic neutrophils can directly and indirectly recruit more neutrophils and other phagocytes, including macrophages and dendritic cells (DCs) [2]. In addition, a variety of infected host cells, such as fibroblasts, endothelial cells and epithelial cells, secrete phagocyte chemoattractants [2,3].

Observing the migration of phagocytes in infected tissue is technically challenging. Numerous in vitro studies of host immune responses to bacteria have been carried out under two-dimensional (2D) co-culture of phagocytes and bacteria, although in tissues, these exist and interact under three-dimensional (3D) conditions. Investigation of cell functions such as phagocytosis and cell migration requires accurate mimicry of the in vivo microenvironment. The migration rate significantly relies on the chemoattractant gradient in tissue. The transwell-based (Boyden chamber) assay is widely used to assess cell migration; however, it provides a bulk end-point measurement and does not measure directionality of cell migration [4]. The assay is not appropriate for assessing migration of cells to a variety of bacteria because non-adherent bacteria grow as a suspension culture in the bottom chamber and can float, adhere, or migrate freely to the upper chamber through the permeable filter of transwell, thus inhibiting chemoattractant gradient formation. Microfluidic devices have been used to study the migration of phagocytes in stable chemokine gradients [5]. A previously developed DC migration assay in fibronectin-coated micro-channels is complementary to more complex 3D migration systems [6,7]. In addition, it has been reported that DCs and T cells readily migrate through the pores in composite macroporous poly(ethylene glycol) hydrogel scaffolds infused with collagen [8]. Recently, Huh et al. developed a microfluidic lung-on-a-chip system consisting of a human alveolar cell layer and a human pulmonary microvascular endothelial cell layer separated by a porous membrane made of poly(dimethylsiloxane) (PDMS) and demonstrated that this system mimics the innate cellular response of bacteria to pulmonary infection [9].

Phagocytes continue to migrate through the extracellular matrix (ECM), a 3D fiber mesh, until they reach an infected or inflamed tissue. Nanofibrous scaffold mimics the configuration of native ECM of tissues [10]. For optimal cellular infiltration, we developed a hybrid electrospun poly(ε-caprolactone) (PCL)-nanofibrous membrane (NM) (PCL-NM) consisting of nano- as well as submicron-scale fibers and we used the PCL-NM to mimic the naturally occurring crosstalk between immune and cancer cells [11]. Additionally, we demonstrated that neutrophils migrate against interleukin (IL)-8 in an electrospun PCL-NM [12]. PCL is a Food and Drug Administration (FDA)-approved biocompatible and biodegradable aliphatic polyester [13]. Furthermore, DCs cultured on PCL nanofibers show a baseline inactive form [11], indicating that PCL is a suitable material for immune cell culture. Thus, we used electrospun PCL-NM to mimic the 3D fibrillar environment in studying the inflammatory response to bacterial infection.

*S. aureus* is a leading cause of human bacterial diseases, including skin and soft tissue infections, food-borne illness, toxic shock syndrome and sepsis. A number of 3D in vitro culture systems have been developed to study the interactions between bacteria and cells [14]. *S. aureus* infection in skin tissue and bone has been studied using 3D co-culture models [15,16]. Ding et al. demonstrated that macrophages can transmigrate across a polyethylene terephthalate membrane to reach the epithelial cell layer cultured with *S. aureus* [17]. In the present study, we used the in vitro infection system to analyze the early events associated with *S. aureus* infection of phagocytes. Cells in 3D cell culture form multilayers of cells, whereas cells grown in 2D form a monolayer. In this study, we designed a chemotaxis assay system using PCL-NM-based two layers, attempting to mimic inflammatory microenvironment, to evaluate early recruitment of phagocytes induced by *S. aureus*. The PCL-NM-based 3D co-culture system can be used to evaluate in vivo-mimicking bacteria-host interactions and immune responses.

## 2. Materials and Methods

### 2.1. Materials

PCL (*M*_n_ = 700,000–900,000), chloroform, PKH26, PKH67, 4-(2-hydroxyethyl)-1-piperazineethanesulfonic acid (HEPES), lipopolysaccharide (LPS), thioglycollate, dimethyl sulfoxide (DMSO) and 4′,6-diamidino-2-phenylindole (DAPI) were purchased from Sigma-Aldrich (St. Louis, MO, USA). Dulbecco’s modified Eagle’s medium (DMEM), RPMI-1640 medium, fetal bovine serum (FBS), penicillin/streptomycin, glutamine and 0.05% trypsin-ethylenediaminetetraacetic acid (EDTA) were purchased from Gibco (Rockville, MD, USA). Granulocyte macrophage-colony stimulating factor (GM-CSF), M-CSF, IL-4 and antibodies against CD11, Ly6G and F4/80 were obtained from R&D Systems Inc. (Minneapolis, MN, USA). Boc-Met-Leu-Phe (Boc-MLF) and Trp-Arg-Trp-Trp-Trp-Trp-NH_2_ (WRW4) were obtained from Tocris bioscience (Bristol, UK). CellTracker Red CMTPX dye was purchased from Molecular Probes, Inc. (Eugene, OR, USA). PDMS was purchased from Dow-Corning Korea, Inc. (Seoul, Korea).

### 2.2. Electrospinning and Fabrication of the PCL Nanofibers

Porous PCL nanofibers were prepared according to the procedure published earlier [11,18]. Briefly, the polymer for electrospinning was dissolved in 99.5% pure chloroform at a concentration of 8.8 wt% and stirred for 5 h to obtain a homogeneous solution. PCL-NMs were fabricated by electrospinning (NanoNC, Seoul, Korea). Two-nozzle spinnerets were used with an average flow rate of approximately 8 µL/min, using a syringe pump. Each nozzle had an inner diameter of approximately 210 µm (27G). Nanofibers were collected onto a rotating metallic mandrel at 100 rpm at ambient temperature for 4 h. The nozzle tip-to-collector distance was set at 20 cm, with an electrical potential of 17.5 kV from the grounded collector plate. An aluminum plate was used for fabricating conventionally electrospun PCL-NM. Membrane thickness was measured using a high-precision caliper (Mitsutoyo Co., Kawasaki, Japan). The morphology of electrospun fibers was observed using scanning electron microscopy (SEM) with a model SEM4500 (Sec, Suwon, Korea). The electrospun PCL-NMs were sterilized by soaking in a solution of 70% ethanol for 12 h and dried under UV exposure for 12 h.

### 2.3. Preparation of Phagocytes from Bone Marrow

BALB/c mice were maintained in accordance with institutional animal care and use guidelines. All experimental procedures involving mice were performed according to protocols approved by the Committee on the Ethics of Animal Experiments of the Ajou University Medical Center (Permit Number: 2015–0028). Bone marrow (BM) cells were harvested from the femurs and tibias of the mice and BM-derived neutrophils were purified using anti-Ly6G antibody-bound microbeads [19]. BM-derived DCs were obtained from BM precursors, as described previously [20]. Briefly, BM cells plated in complete RPMI-1640 containing recombinant murine GM-CSF (20 ng/mL) plus recombinant murine IL-4 (20 ng/mL). On the third day of incubation, the same concentration of IL-4 and GM-CSF was added and half of the DC culture medium was refreshed. All BM cells were harvested on day 7 of incubation. By immunomagnetic selection of CD11c^+^ cells, BMDCs were purified to >90% using CD11c Microbeads Ultrapure (Miltenyi Biotec, Bergisch-Gladbach, Germany). BM-derived macrophages were obtained by culturing of mouse BM cells with recombinant murine M-CSF (20 ng/mL) for 7 days [21]. BM-derived DCs and macrophages were purified using anti-CD11c and anti-F4/80 antibody-bound microbeads, respectively. The purity of neutrophils, DCs and macrophages was verified to be >90% by flow cytometry using surface markers, Ly6G, CD11c and F4/80, respectively.

### 2.4. Preparation of Peritoneal Neutrophils and Macrophages 

BALB/c mice were treated with 3% thioglycollate in distilled water (intraperitoneal injection) for 6 h and 3 days to purify peritoneal neutrophils and macrophages, respectively, which were isolated by lavage with phosphate-buffered saline (PBS) and purified with anti-Ly6G- and anti-F4/80 antibody-bound microbeads, respectively.

### 2.5. MLE-12 Cell Culture

MLE-12 mouse lung epithelial type II cells were purchased from American Type Culture Collection (ATCC) (Manassas, VA, USA) and cultured in ATCC-formulated DMEM supplemented with 10% FBS, 1% penicillin/streptomycin at 37 °C in 5% CO_2_ incubator. MLE-12 cells were subcultured every 3–4 days and cells at passages 6–10 were used herein.

### 2.6. Bacterial Culture and Adhesion Assay

*S. aureus* strain RN4220 was grown to mid-log phase in Luria-Bertani (LB) broth, washed and resuspended in PBS [22]. The bacterial concentration was estimated by determining colony forming units (CFUs) and the optical density at 600 nm (OD_600_) using a spectrophotometer. The bacterial suspension was diluted to an OD_600_ of 0.1. Bacteria were also stained with PKH67 Green and PKH26 Red Fluorescent Cell Linker Kits (Sigma) and washed with PBS three times. PDMS (100 µL) was poured in an 8-well plate (SPL, Seoul, Korea) and kept on a slide warmer at 100 °C for 3 min. The PCL-NM (1.1 cm × 1.3 cm) was attached to the surface of gel-state PDMS on an 8-well plate and immersed in DMEM (700 µL) for 6 h to increase cell adhesion at 37 °C. For bacterial culture in the PCL-NM, a bacteria suspension (1 × 10^7^ CFU/200 µL) was seeded on the surface of a wet PCL-NM. After incubation at 37 °C and under 5% CO_2_ for the indicated times to allow the bacteria to settle and attach to the nanofibers, the PCL-NMs were gently rinsed thrice with PBS to remove not-adherent bacteria.

### 2.7. Laser Confocal Microscopy 

Fluorescence-labeled phagocytes and *S. aureus* on a culture dish and in the PCL-NM were observed using a K1 confocal microscope (Nanoscope, Daejeon, Korea) at the 3D immune system imaging core facility of Ajou University. Images was analyzed using the ImageJ software.

### 2.8. SEM

Phagocytes and/or bacteria cultured in the PCL-NM were affixed to aluminum mounts with double-sided carbon tape (SPI Supplies Inc., Seoul, Korea) and coated with gold-sputter. Cell structure and nanofiber morphology were observed using SEM.

### 2.9. FACS Analysis of Phagocytosis

Neutrophils, macrophages and DCs were labeled with the PKH67 fluorescent dye and *S. aureus* were labeled with the PKH26 fluorescent dye. The cells were washed thrice with PBS. The labeled phagocytes (1 × 10^5^) were allowed to adhere to the culture dish for 1 h and cultured with labeled *S. aureus* (1 × 10^6^ CFU) in RPMI 1640 medium (200 µL) in the absence or presence of 5% FBS at 37 °C in a humidified atmosphere containing 5% CO_2_. For the phagocytic assay in the PCL-NM, labeled phagocytes (1 × 10^5^) were top-seeded on the PCL-NM surface and incubated for 1 h; then, the bacterial suspension (1 × 10^6^ CFU/200 µL) was added. For the assay with formyl peptide receptor (FPR) inhibitors, Boc-MLF and WRW4 were dissolved in DMSO and PBS, respectively, and 0.1% DMSO solution was used as a control. Phagocytes and bacteria were detached from the PCL-NM by soaking in trypsin-EDTA solution for 5 min. The percentage of PKH26-stained cells among total PKH67-stained phagocytes was analyzed using flow cytometry.

### 2.10. Live Imaging of Phagocytosis 

The dynamic interactions between *S. aureus* and phagocytes were visualized using a K1 confocal microscopy. Neutrophils, macrophages and DCs (1 × 10^5^) were labeled with CellTracker and *S. aureus* cells (1 × 10^7^ CFU) were labeled with PKH67 fluorescent dye. Labeled phagocytes were top-seeded on PCL-NMs in 3D culture and on culture dishes in 2D culture. After a 4 h incubation of phagocytes (1 × 10^5^/100 µL) for cell attachment to the culture dish or PCL-NM, *S. aureus* suspension (1 × 10^6^ CFU/100 µL) in RPMI-1640 culture media was added to the labeled phagocytes. Time-lapse videos of phagocytes engulfing bacteria were generated by taking pictures every 60 s for 1 h.

### 2.11. Migration Assay in PCL-NM-Based Two-Layer System

We assembled a two-layer system of two PCL-NMs for cell migration, as shown in supplementary material Figure S1, in a modified Transwell chamber [18]. To construct the upper PCL-NM in the apical chamber, the polycarbonate filter of a Transwell insert was removed and replaced with a 5-mm-diameter circular section of PCL-NM, which were attached with gel-state PDMS. Neutrophils, macrophages and DCs were labeled with PKH26 dye and 10 µL of cell suspension (1 × 10^5^) was seeded onto the PCL-NM in the apical chamber. After 4 h, the scaffold was washed with PBS. For the lower PCL-NM layer, PDMS (220 µL) was poured in the lower cultivation well to maintain constant distance from upper surface of the lower membrane to the lower surface of the upper membrane and a PCL-NM was attached on top of the surface of the gel-state PDMS. *S. aureus* suspension (1 × 10^7^ CFU) was added to the surface of the PCL-NM in the basal chamber and bacteria were allowed to adhere for 2 h. The phagocyte- and *S. aureus*-seeded membranes were then assembled layer-by-layer by placing the insert in the well. The chamber contained 600 µL of medium and phagocytes were allowed to migrate at 37 °C in a humidified CO_2_ atmosphere. Migrated PKH26-labeled cells in the lower PCL-NM layer were counted under a confocal microscope in three random fields at a magnification of 100×. A lower PCL-NM layer without bacteria served as a control to evaluate migration of the phagocytes.

### 2.12. Cytokine Assay

Secretion of cytokines and chemokines was determined using a Proteome Profiler Mouse Cytokine Array Kit (R&D Systems). Neutrophils and lung epithelial cells (1 × 10^5^) in the presence or absence of *S. aureus* (1 × 10^7^ CFU) were cultured on a culture dish and in the PCL-NM. After culture for the indicated times, supernatants were taken and assayed as described in the user manual. Chemiluminescence signal density was quantified with ImageJ. IL-8 levels were measured by ELISA (R&D Systems) according to manufacturer’s instruction.

### 2.13. Statistical Analysis

The results are presented as means ± standard deviations (SDs). Student’s *t*-test was used to compare the means of paired or unpaired samples. A *p*-value of <0.05 was considered significant.

## 3. Results

### 3.1. Culture of S. aureus and Phagocytes in Electrospun PCL-NM

The PCL-NM had an average thickness of 57.0 ± 5.5 µm (*n* = 10). The ultrastructure of PCL-NM was analyzed by SEM to assess the fiber size, fiber uniformity and surface topography [11]. The electrospun samples were homogeneous and the majority of fiber diameters in the membranes was between 400 nm and 2 µm (data not shown). The most frequent fiber diameter was between 200 nm and 600 nm, but substantial microfibers with a diameter of 1–10 µm were deposited in the PCL-NM (data not shown) [18]. Thus, microfibers in the PCL-NM introduced larger pores than nanofibers. The mercury intrusion data for electrospun PCL-NM showed pores with a diameter of approximately 10–100 µm (data not shown) [11]. The large pores in the nano-submicron PCL-NM were expected to allow phagocytes to infiltrate deep into the membrane [11].

**Figure 1:**
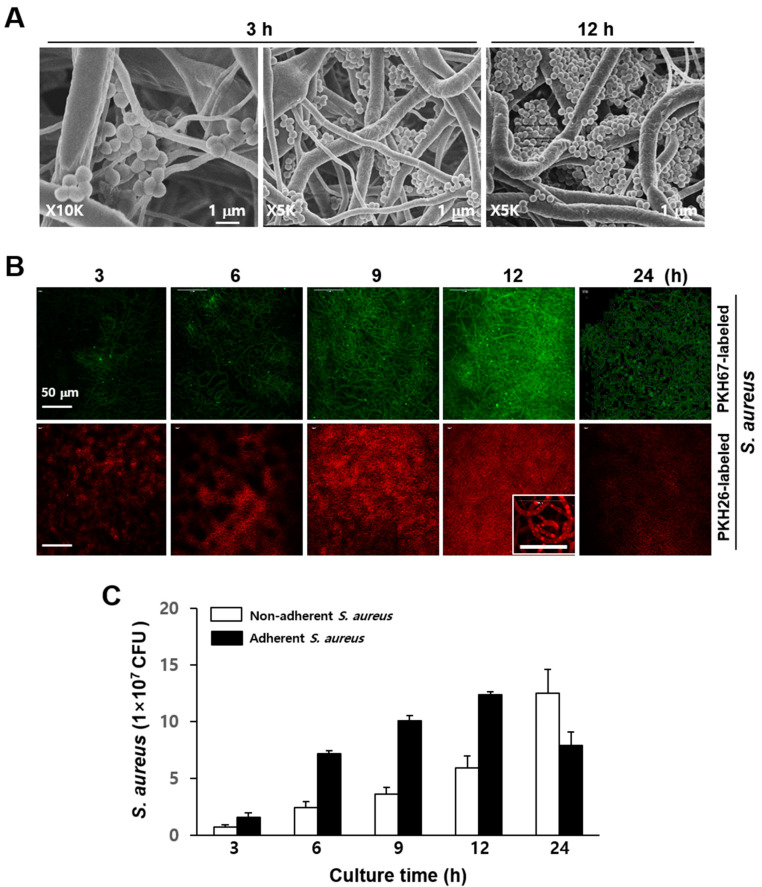
Adhesion of *S. aureus* to the PCL-NM. (**A**) *S. aureus* (1 × 10^7^ CFU) were top-seeded on the PCL-NM for the indicated times and adhesion of *S. aureus* to the nanofibers was evaluated by SEM. Images are representative of three independent experiments. (**B**) PKH67- and PKH26-labeled *S. aureus* (1 × 10^7^ CFU) were top-seeded on the PCL-NM and cultured for the indicated times (*n* = 3). The adhesion and proliferation of fluorescence-labeled *S. aureus* in the PCL-NM were evaluated by confocal microscopy. High magnification image (×1000) in the box shows numerous spots along the nanofibers. (**C**) *S. aureus* (1 × 10^7^ CFU/200 µL) were cultured in the PCL-NM for the indicated times. Non-adherent bacteria were gently recovered from culture media (50 µL) and adherent bacteria on nanofibers were harvested by vigorous pipetting after the addition of culture media (100 µL) to the PCL-NM. The numbers of non-adherent and adherent bacteria in the PCL-NM were estimated by determining CFUs. Data are presented as means ± SDs (*n* = 3).

First, we evaluated the attachment of the bacteria to the nanofibers by SEM in an experiment using bacteria alone. *S. aureus* were top-seeded on a PCL-NM and allowed to infiltrate. *S. aureus* are Gram-positive round cells of 0.5–1 µm diameter. As shown in Figure 1A, the bacteria were well distributed throughout the membrane and attached along the nanofiber surfaces. Small bacterial agglomerates were seen between two or more fibers in areas of fiber cross over. Bacteria cultured for 12 h formed aggregates inside the PCL-NM. Bacterial viability and the proliferation of PKH-labeled and unlabeled cells are identical, indicating that PKH fluorescent dyes are nontoxic to bacteria [23]. In addition, the fluorescence intensity of PKH-labeled bacteria remains constant during the lag phase, whereas replication results in a 50% decrease in fluorescence with each cell division [23]. As shown in Figure 1B, the density of PKH-labeled bacteria exhibiting fluorescence in the PCL-NM increased up to 12 h, but then decreased at 24 h. Next, we quantified bacteria after detachment from nanofibers. The numbers of *S. aureus* adhering to the nanofibers increased in a time-dependent manner up to 12 h, but then decreased at 24 h (Figure 1C).

**Figure 2:**
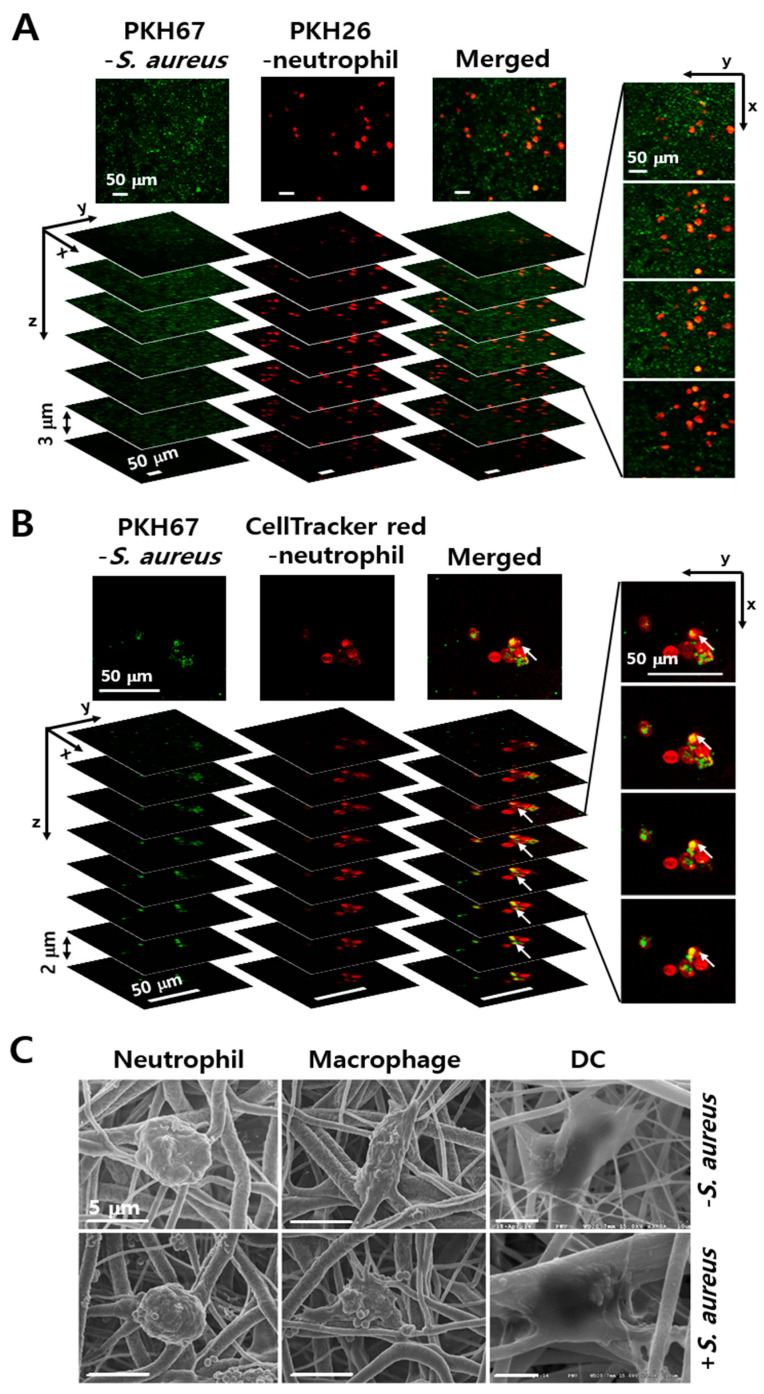
Co-culture of phagocytes and *S. aureus* in the PCL-NM. (**A**,**B**) Peritoneal neutrophils were labeled with PKH26 (**A**) and CellTracker red (**B**). The labeled neutrophils (1 × 10^5^) and PKH67-labeled *S. aureus* (1 × 10^6^ CFU) were top-seeded on the PCL-NM and cultured for 1 h in the membranes (*n* = 3). Co-localization of neutrophils and bacteria was evaluated by focus-stacking images of the PCL-NM. Images of 3 µm (**A**) and 2 µm (**B**) in thickness from the top to bottom are shown. Arrows indicate green-fluorescent spots inside of red-fluorescent areas. (**C**) Neutrophils, macrophages and DCs (1 × 10^5^) were top-seeded on the PCL-NM and cultured with (+) or without (−) *S. aureus* (1 × 10^6^ CFU) for 1 h in the PCL-NM. The localization of *S. aureus* and phagocytes in the PCL-NM was evaluated by SEM. Data are representative of three independent experiments.

In comparison, the numbers of non-adherent *S. aureus* increased up to 24 h. These results indicated that the bacteria could proliferate on the fibers, producing agglomerates, but are not able to maintain large colonies in the PCL-NM after prolonged culture, possibly because of the small diameter (400 nm–2 µm) of the nanofibers. To rule out the toxic effect of PCL-NM, PCL-NM alone was soaked in culture media for 24 h and bacteria were cultured in the media for 24 h. We confirmed that the growth of *S. aureus* was not affected (data not shown). The spreading and infiltration of bacteria and neutrophils in the PCL-NM were evaluated 1 h after cells were seeded on the membranes. A focus-stacking analysis of confocal microscopy images captured at 3 µm intervals showed that fluorescent-labeled bacteria and neutrophils infiltrated the membrane from the upper surface to a depth 15–30 µm (Figure 2A). Bacteria and neutrophils were homogeneously distributed at a similar level inside the PCL-NM. Moreover, the green fluorescence of bacteria co-localized with red fluorescence of neutrophils, indicating internalization of the bacteria by the neutrophils (Figure 2B). In addition, co-localization of bacteria with neutrophils, macrophages and DCs cultured in the PCL-NM was confirmed by SEM (Figure 2C). PCL nanofibers have various fiber diameters and pore sizes, which depend on polymer concentration, flow rate and others. Fabrication of PCL nanofibers through electrospinning facilitated PCL-NM to allow the infiltration of bacteria and phagocytes. Therefore, the PCL-NM can be used for the 3D culture of bacteria and phagocytes.

### 3.2. Engulfment of S. aureus by Phagocytes in the PCL-NM 

In live cell imaging analysis, PKH67-labeled *S. aureus* were caught and ingested by CellTracker red CMTPX-labeled neutrophils and DCs in 2D as well as 3D culture. In monolayer co-culture, neutrophils adhered to the surface of culture dish but did not show chemotactic migration toward bacteria because the latter were floating around in the media (Supplementary Video S1). On the other hand, the DCs adherent on the plastic surface showed dynamic changes in intracellular red dye, which we first hypothesized to represent cell spreading to pick up bacteria; however, careful observation learned that the changes in dye levels were located inside the cytoplasm and did not represent dynamic cell extensions (Supplementary Video S2). In contrast, single-cell tracking in the PCL-NM revealed changes in neutrophil morphology and migration, with directional movement toward the bacteria (Supplementary Video S3). Neutrophils extended long protrusions of cell body along the nanofibers and then retracted the protrusion, indicating transition from the spreading to the migratory state. When assuming a cell size of 10 µm, the leading edge elongated several folds. Time-lapse microscopy clearly showed CellTracker-labeled DCs crawling along the nanofibers to nearby green-fluorescent PKH67-labeled *S. aureus*, with high motility to pick up the bacteria (Supplementary Video S4). These results suggested that co-culture of bacteria and phagocytes in the PCL-NM allows assessing phagocytosis of bacteria in 3D condition.

### 3.3. Differential Rate of Phagocytosis in 2D and 3D Culture Conditions

**Figure 3:**
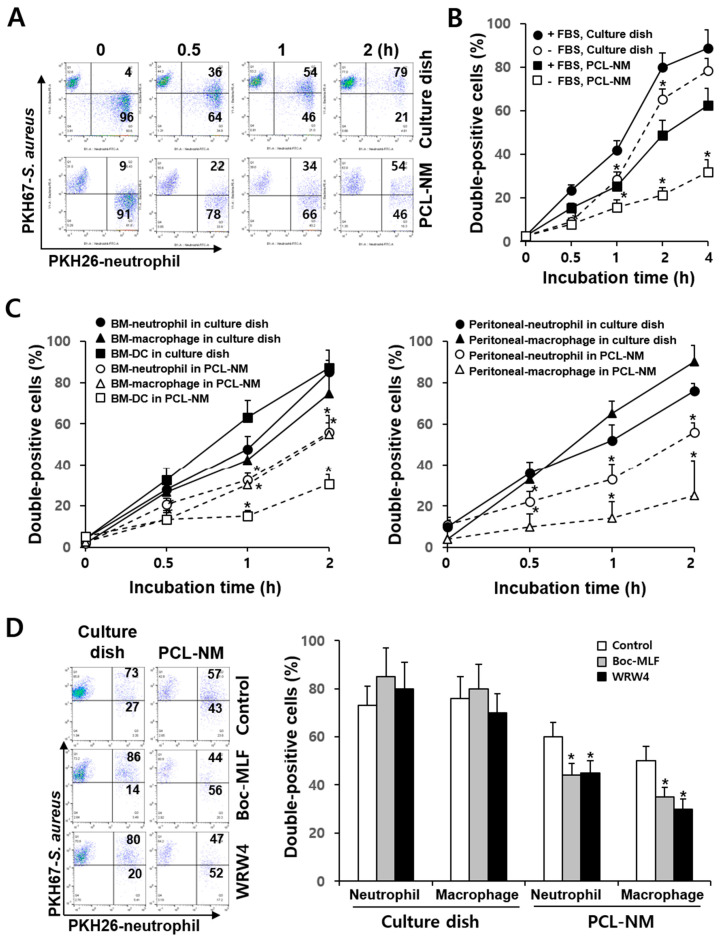
Phagocytosis of *S. aureus* by neutrophils, macrophages and DCs in the PCL-NM. (**A**) PKH67-labeled *S. aureus* (1 × 10^6^ CFU) was cultured with PKH26-labeled BM-derived neutrophils (1 × 10^5^) for the indicated times on a culture dish and in the PCL-NM and double-positive cells among PKH26-positive cells were detected by flow cytometry (*n* = 4). Numbers represent the percentages of cells within the indicated gates of PKH26-labeled neutrophils. (**B**) BM-derived neutrophils were stained with PKH26 and cultured with PKH67-labeled *S. aureus* (1 × 10^6^ CFU) in the presence (+FBS) or absence (−FBS) of 5% FBS for the indicated times on a culture dish and in the PCL-NM. Rates of phagocytosis were evaluated by counting double-positive cells among PKH26-labeled phagocytes. Data are presented as means ± SDs (*n* = 3). Asterisks (*) denote significant differences compared to culture with FBS (*p* < 0.05). (**C**) BM- and peritoneal-derived phagocytes were prepared as described in the Materials and methods. BM- and peritoneal-derived neutrophils, macrophages and DCs (1 × 10^5^) were stained with PKH26 and cultured with PKH67-stained *S. aureus* (1 × 10^6^ CFU) for the indicated times in the presence of 5% FBS on a culture dish and in the PCL-NM. Rates of phagocytosis were evaluated by counting double-positive cells among PKH26-labeled neutrophils, macrophages and DCs. Data are presented as means ± SDs (*n* = 4). Asterisks (*) denote significant differences compared to culture on a culture dish (*p* < 0.05). (**D**) Phagocytosis of *S. aureus* (1 × 10^6^ CFU) by BM-derived neutrophils and macrophages (1 × 10^5^) in the PCL-NM containing 5% FBS was measured in the presence of 0.1% DMSO (Control), Boc-MLF (20 µM) and WRW4 (4 µM) (*n* = 4). Numbers in the left panel represent the percentage of cells within the indicated gates of PKH26-labeled neutrophils. Data are presented as means ± SDs. Asterisks (*) denote significant differences compared to the control (*p* < 0.05).

It has been demonstrated that there is a significant difference in phagocytosis of *S. aureus* between adherent and suspended neutrophils; the phagocytosis by adherent neutrophils is largely unaffected by opsonization with serum [24]. We compared the rate of phagocytosis of live bacteria between phagocytes cultured in 2D and 3D conditions. Flow cytometry can be used for the fluorescence quantitation of the phagocytosis of labeled bacteria [23]. PKH26-labeled neutrophils were incubated for 1 h on a culture dish and in the PCL-NM and cultured with PKH67-labeled *S. aureus* for 2 h. Flow cytometry assays showed that double positive-stained cells correspond to neutrophils that internalized bacteria and the fluorescence intensity of PKH67-labeled *S. aureus* remained constant during 2 h of culture in the presence of neutrophils on a culture dish and in the PCL-NM (Figure 3A). Thus, we can rule out the possibility that fluorescence was lost during bacterial culture and that the rate of phagocytosis was underestimated in 2D and 3D conditions. The uptake of bacteria by BM-derived neutrophils occurred in a time-dependent manner on culture dishes and in the PCL-NMs containing 5% FBS (Figure 3B). Next, we measured the phagocytosis rate on culture dishes and in the PCL-NMs containing serum-free media. The phagocytic rates of neutrophils on the culture dish and in the PCL-NM were significantly lower in serum-free media than in FBS-containing media. The reduction in the rate of phagocytosis in serum-free media was greater in the PCL-NMs than on culture dishes. The levels of phagocytosis 4 h after the co-culture of neutrophils and bacteria increased to 80–90% on culture dishes containing serum-free media and FBS-containing media. In contrast, the uptake of bacteria by neutrophils after 4 h of bacterial and neutrophil co-culture was significantly lower in serum-free condition than in FBS-containing media in the PCL-NM (32.0 ± 5.6% vs. 62.3 ± 8.0%, *p* < 0.05). The uptake of bacteria by BM-derived and peritoneal phagocytes in the presence of FBS occurred in a time-dependent manner (Figure 3C). Half of total peritoneal neutrophils (50.3 ± 5.1%) and the majority of peritoneal neutrophils (79.5 ± 6.5%) were double positively stained after 1 h and 2 h of 2D culture, respectively. In contrast, in the PCL-NM, the number of BM-derived and peritoneal neutrophils that had phagocytosed bacteria was lower than that in 2D culture. BM-derived macrophages and DCs rapidly phagocytosed bacteria, but the phagocytic rate and ability in the PCL-NM were lower than those on the culture dish.

### 3.4. Effects of FPR Inhibitors on the Phagocytosis of S. aureus in 2D and 3D Culture Conditions

Phagocytes sense chemotactic gradients produced by bacteria, triggering phagocytosis. The bacterial peptide, fMLP is a highly potent leukocyte chemoattractant and chemotactic activity of phagocytes is mediated by FPR [25]. We next compared the effect of Boc-MLF and WRW4, selective FPR1 and FPR2 inhibitors in murine neutrophils [26], on phagocytosis by neutrophils and macrophages in 2D and 3D culture conditions. Boc-MLF and WRW4 decreased the phagocytic rate of neutrophils in the PCL-NM when compared to that of neutrophils grown in control condition (44 ± 5% and 45 ± 6% vs. 60 ± 8%, respectively, *p* < 0.05) (Figure 3D). Similar results were obtained for macrophages treated with Boc-MLF and WRW4 when compared to macrophages without inhibitors (35 ± 4% and 30 ± 3% vs. 50 ± 7%, respectively, *p* < 0.05). In contrast, no inhibition of phagocytosis was observed when FPR inhibitors were added to co-culture of *S. aureus* and neutrophils or macrophages in 2D culture. These results suggested that phagocyte migration plays a role in phagocytosis in 3D, but not in 2D culture.

### 3.5. 3D Migration of Phagocytes to S. aureus in PCL-NM-Based Two-Layer Culture System

**Figure 4:**
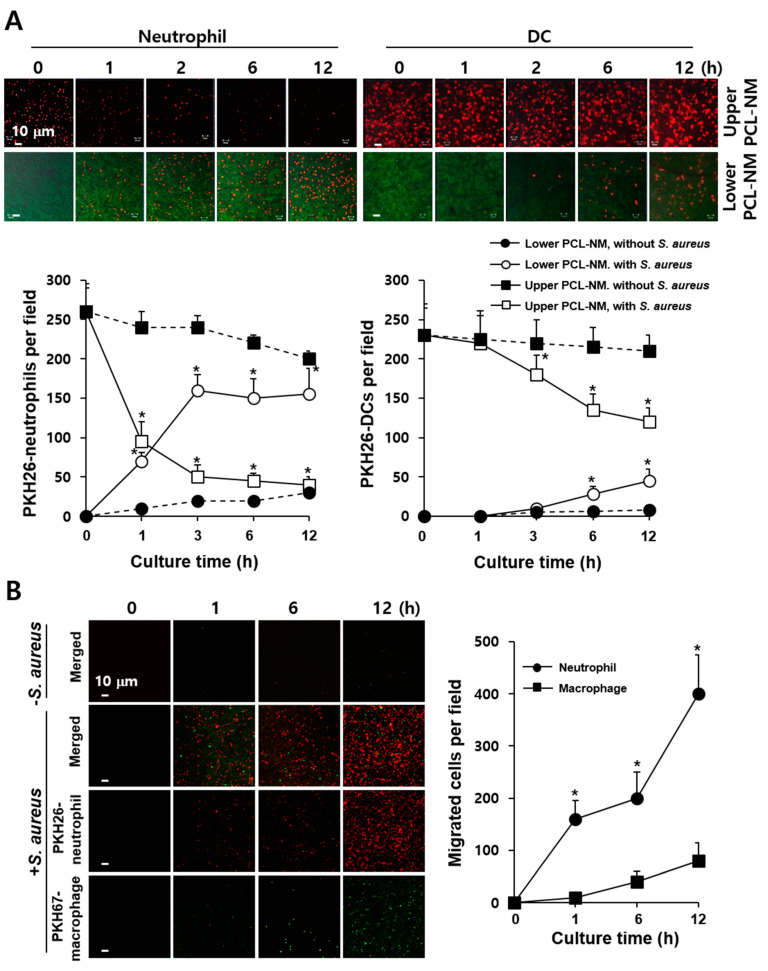
Migration of phagocytes to *S. aureus* in the PCL-NM-based two-layer culture system. (**A**) PKH26-labeled BM-derived neutrophils and DCs (1 × 10^5^) and PKH67-labeled *S. aureus* (1 × 10^7^ CFU) were seeded and cultured in the upper and lower PCL-NMs, respectively, for the indicated times (*n* = 4). The graphs depict the numbers of red-fluorescent spots detected in the upper (■, □) and lower (●, ○) PCL-NMs when bacteria were absent (●, ■) or present (○, □) in the lower PCL-NM. Data are presented as means ± SDs (*n* = 4). Asterisks (*) denote significant differences compared to culture without bacteria (*p* < 0.05). (**B**) PKH26-labeled peritoneal neutrophils (1 × 10^5^) and PKH67-labeled peritoneal macrophages (1 × 10^5^) were seeded in the upper PCL-NM and *S. aureus* (1 × 10^7^ CFU) was seeded in the lower PCL-NM. Phagocytes in the upper PCL-NM were cultured in the presence (+ *S. aureus*) or absence (−*S. aureus*) of bacteria in the lower PCL-NM for the indicated times (*n* = 3). The graphs depict the numbers of red- (Neutrophil) and green- (Macrophage) fluorescent spots detected in the lower PCL-NM containing the bacteria. Data are represented as means ± SDs (*n* = 3). Asterisks (*) denote significant differences compared to culture with macrophages (*p* < 0.05).

As shown in supplementary Figure S1, 3D migration of phagocytes to bacteria was assessed quantitatively using PCL-NM-based two-layer system in a Transwell chamber. PKH26-labeled phagocytes and PKH67-labeled bacteria were cultured in the upper and lower PCL-NM layers, respectively. After indicated times of incubation, the numbers of phagocytes that had migrated from the upper to the lower PCL-NM were counted. As shown in Figure 4A, increasing numbers of neutrophils and DCs were detected in the lower PCL-NM over time, while the numbers of phagocytes in the upper PCL-NM gradually decreased. Neutrophils migrated rapidly in response to *S. aureus*, when compared to DCs. Next, we performed a similar assay, in which equal numbers of PKH26-labeled neutrophils and PKH67-labeled macrophages were top-seeded on the upper PCL-NM and bacteria on the lower PCL-NM. While both macrophages and neutrophils migrated to the lower PCL-NM, the numbers and rates of migration of macrophages were lower than those of neutrophils (Figure 4B).

### 3.6. Neutrophil-Induced Recruitment of More Neutrophils to S. aureus in PCL-NM-Based Two-Layer Culture System

**Figure 5:**
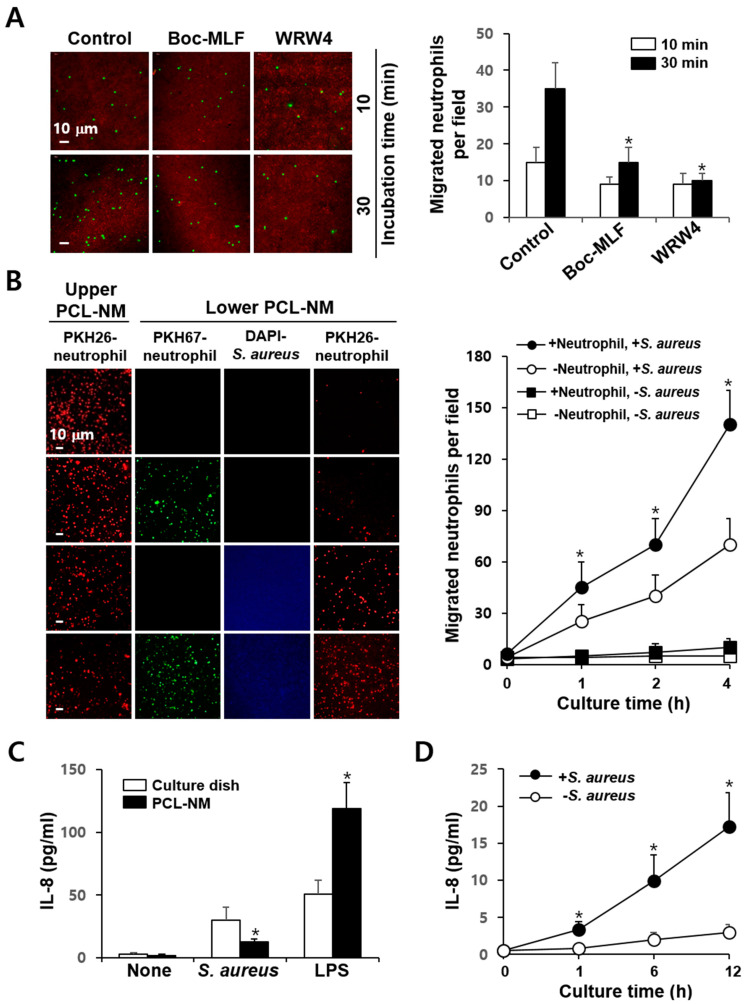
More neutrophils are recruited when *S. aureus* is cultured with neutrophils than when it is cultured alone. (**A**) PKH67-labeled BM-derived neutrophils (1 × 10^5^) were top-seeded and pretreated with 0.1% DMSO (Control), Boc-MLF (20 µM) and WRW4 (4 µM) for 1 h in the upper PCL-NM. The migration rates of control and FRP inhibitor-pretreated neutrophils to the lower PCL-NM containing PKH26-labeled *S. aureus* (1 × 10^7^) were measured for the indicated times in the presence of 0.1% DMSO (control), Boc-MLF (20 µM) and WRW4 (4 µM) (*n* = 4). Green-fluorescent spots in the lower PCL-NM were counted. Asterisks (*) denote significant differences compared to the control (*p* < 0.05). (**B**) PKH26-labeled neutrophils (1 × 10^5^) in the upper PCL-NM were cultured with or without PKH67-labeled neutrophils (1 × 10^5^) and/or DAPI-stained *S. aureus* (1 × 10^7^ CFU) in the lower PCL-NM (*n* = 4). Red-fluorescent spots were detected in the lower PCL-NM. The graphs depict the numbers of red-fluorescent spots detected in the lower PCL-NM in the absence (○, □) or presence (●, ■) of neutrophils with (○, ●) or without (□, ■) *S. aureus*. Data are presented as means ± SDs (*n* = 4). Asterisks (*) denote significant differences compared to the culture condition lacking neutrophil with *S. aureus* in the lower PCL-NM (*p* < 0.05). (**C**) Neutrophils (1 × 10^5^) were cultured in media alone (None), with *S. aureus* (1 × 10^7^ CFU) and with LPS (1 µg/mL) for 12 h on a culture dish and in the PCL-NM. Data are presented as means ± SDs (*n* = 3). Asterisks (*) denote significant differences compared to untreated control (*p* < 0.05). (**D**) Neutrophils (1 × 10^5^) were cultured with (+) or without (−) *S. aureus* (1 × 10^7^ CFU) for the indicated times in the PCL-NM. IL-8 concentrations in culture media were measured by ELISA. Data are presented as means ± SDs (*n* = 4). Asterisks (*) denote significant differences compared to culture without *S. aureus* (*p* < 0.05).

Neutrophil migration is mediated by chemokines derived from bacteria and phagocytes. When PKH67-labeled neutrophils were pretreated with FPR inhibitors and then cultured with PKH26-stained bacteria in the presence of the inhibitors in the PCL-NM-based two-layer system, the numbers of PKH67-positive neutrophils in the lower PCL-NM were significantly decreased when compared to those of untreated neutrophils (Figure 5A). The effects of FPR inhibitors on migration were only obvious in the first hour after co-culture of neutrophils in the upper PCL-NM and bacteria in the lower PCL-NM, indicating that the early phase of migration is mediated by bacteria-derived chemoattractants, such as fMLP. It is possible that the secretion of chemokines by neutrophils migrated from the upper to the lower PCL-NM may trigger a later, second phase of neutrophil migration. It has been shown that DAPI staining does not impact the growth of *Listeria monocytogenes* in culture media and macrophages [27]. Thus, we measured the migration rate of PKH26-labeled neutrophils in the upper PCL-NM using cultures of PKH67-labeled neutrophils and/or DAPI-stained bacteria in the lower PCL-NM. More PKH26-labeled neutrophils were observed in the lower PCL-NM containing both *S. aureus* and neutrophils than in the lower PCL-NM containing *S. aureus* or neutrophils alone (Figure 5B). Neutrophils cultured with bacteria in PCL-NM as well as in culture dish secreted IL-8 into the medium (Figure 5C). The concentrations of IL-8 secreted by neutrophils cultured with bacteria in the PCL-NM were lower than those in 2D culture, but increased in a time-dependent manner (Figure 5D). These results indicated that neutrophil-derived chemokines in the lower compartment, such as IL-8, enhance the migration of neutrophils from the upper to the lower PCL-NM.

### 3.7. Engulfment of Neutrophils by DCs in the Presence of S. aureus in PCL-NM-Based Co-Culture Condition

**Figure 6:**
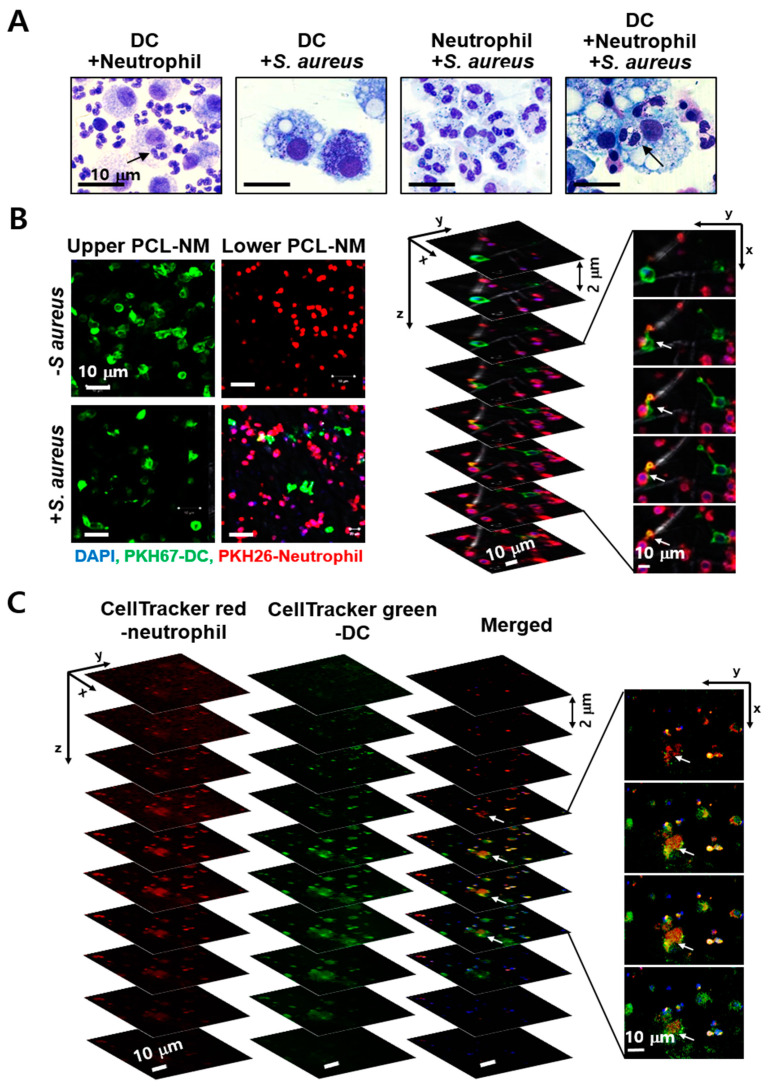
Engulfment of neutrophils by DCs in 2D and 3D culture conditions. (**A**) DCs (2 × 10^5^) and/or neutrophils (1 × 10^6^) were cultured with or without *S. aureus* (5 × 10^6^ CFU) for 6 h in culture dishes and stained with Giemsa solution (*n* = 3). Arrows indicate live neutrophils inside of DCs. (**B**) PKH26-labeled neutrophils (2 × 10^6^) were cultured for 2 h with or without DAPI-stained *S. aureus* (2 × 10^7^ CFU) in the lower PCL-NM. PKH67-labeled DCs (2 × 10^5^) in the upper PCL-NM were cultured for 4 h with bacteria and neutrophils in the lower PCL-NM (*n* = 3). Green-fluorescent spots were detected in the lower PCL-NM (in the left panel). The right panel depicts focus-stacking images of the lower PCL-NM. (**C**) CellTracker green-labeled DCs in the upper PCL-NM were cultured with CellTracker red-labeled neutrophils and DAPI-stained bacteria as described in figure B (*n* = 3). Arrows indicate red-fluorescent spots inside of green-fluorescent areas in the lower PCL-NM. Images show stacks of 2 µm in thickness from the top to bottom.

Co-culture of DCs and neutrophils showed internalization of live neutrophils by DCs [28]. This study confirmed that DCs engulfed live neutrophils in the presence or absence of *S. aureus* when neutrophils and DCs were co-cultured at a ratio of 10:1 on a culture dish (Figure 6A). However, it is unknown whether the engulfment of viable neutrophils by DCs occurs in bacteria-infected tissues in vivo. Moreover, we cannot rule out the possibility of experimental artifact during co-culture of different types of phagocytes in 2D culture. Thus, we investigated whether DCs in the upper PCL-NM migrate to and are able to uptake bacteria-engulfing neutrophils in the lower PCL-NM using a two-layer culture system. *S. aureus* was labeled with DAPI and cultured with PKH26-labeled neutrophils for 2 h in the lower PCL-NM. Neutrophils and bacteria in the lower PCL-NM were further cultured for 4 h with PKH67-labeled DCs in the upper PCL-NM. As shown in Figure 6B, DCs migrated to the lower PCL-NM containing both neutrophils and bacteria, but did not migrate to the lower PCL-NM harboring neutrophils alone. Focus-stacking images of the lower PCL-NM showed that PKH67-labeled DCs extended dendrites to PKH26-labeled neutrophils. In *z*-stack analysis of confocal microscopic images, the red fluorescence of CellTracker red-labeled neutrophils co-localized with green fluorescence of CellTracker green-labeled DCs, indicating internalization of the neutrophils by the DCs (Figure 6C). This result indicated that DCs migrate to neutrophils phagocytosing bacteria and then engulf neutrophils in 3D in vitro culture.

### 3.8. Migration of Phagocytes to S. aureus–Infected Epithelial Cells in PCL-NM-Based Two-Layer Culture System 

**Figure 7:**
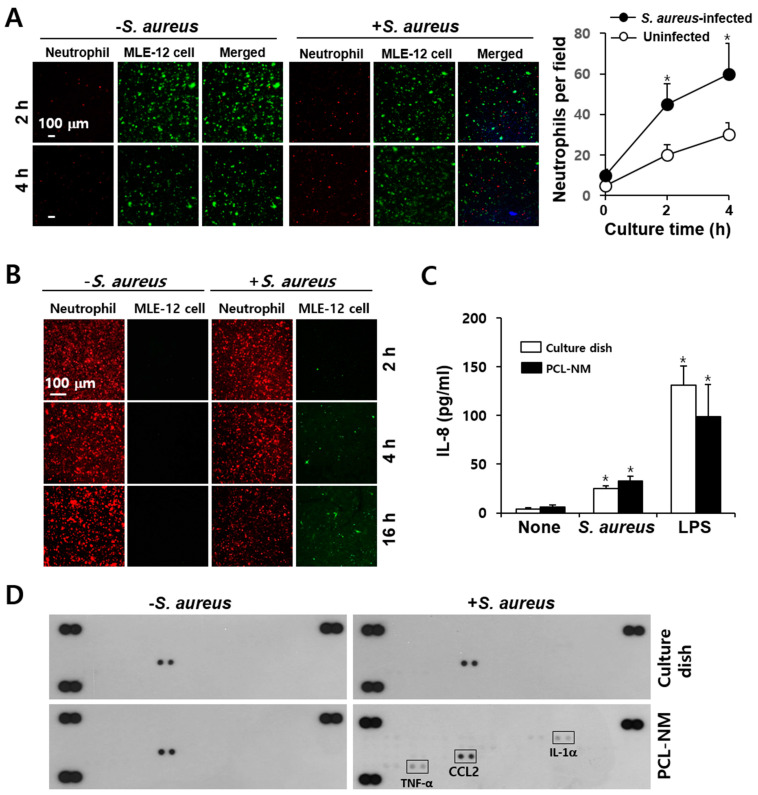
Migration of neutrophils to *S. aureus*-infected epithelial cells in the PCL-NM-based two-layer culture system. (**A**) PKH67-labeled MLE-12 cells (1 × 10^5^) were incubated with or without DAPI-stained *S. aureus* (1 × 10^7^ CFU) for 12 h in the lower PCL-NM and washed with culture media. PKH26-labeled peritoneal neutrophils (1 × 10^5^) were seeded on the upper PCL-NM and cultured for the indicated times with untreated and bacteria-infected MLE-12 in the lower PCL-NM. Red-fluorescent spots were detected in the lower PCL-NM containing PKH67-labeled uninfected and *S. aureus*-infected MLE-12 cells (Epithelial cell) (*n* = 3). The graphs depict the numbers of red-fluorescent spots detected in the lower PCL-NM. Data are presented as means ± SDs (*n* = 3). Asterisks (*) denote significant differences compared to uninfected MLE-12 cells (*p* < 0.05). (**B**) Green-fluorescent spots of MLE-12 cells (Epithelial cell) were detected on the upper PCL-NM containing PKH26-labeled neutrophils (Neutrophil) (*n* = 3). (**C**) MLE-12 cells (1 × 10^5^) were cultured with media alone (None), *S. aureus* (1 × 10^7^ CFU) and LPS (1 µg/mL) for 12 h on a culture dish and in the PCL-NM. The secretion of IL-8 was measured by ELISA. Data are presented as means ± SDs (*n* = 3). Asterisks (*) denote significant differences compared to None (*p* < 0.05). (**D**) MLE-12 cells (1 × 10^5^) were cultured with (+) or without (−) *S. aureus* (1 × 10^7^ CFU) for 12 h on a culture dish and in the PCL-NM. Cytokines and chemokines secreted in the culture media were detected using cytokine and chemokine arrays (*n* = 3).

Fibroblasts, epithelial cells and endothelial cells are potentially important targets for *S. aureus* infection [29]. PKH67-labeled MLE-12 cells and DAPI-stained *S. aureus* were co-cultured for 12 h in the lower PCL-NM layer and then, PKH26-labeled phagocytes were top-seeded on the upper PCL-NM layer. The migration of phagocytes to infected epithelial cells was measured. As shown in Figure 7A, the neutrophils migrated toward the lung epithelial cells when these were infected with *S. aureus*. Immunofluorescence analysis of the upper PCL-NM showed that the numbers of red-fluorescent neutrophils decreased but those of green-fluorescent epithelial cells from the lower PCL-NM increased in a time-dependent manner (Figure 7B). In contrast, *S. aureus*-infected epithelial cells were not detected in the upper PCL-NM when it had not been seeded with neutrophils (data not shown). These results indicated that neutrophils in the upper PCL-NM migrated to the lower PCL-NM and damaged epithelial cells in the lower PCL-NM floated up to the upper PCL-NM. On the basis of these observations, we tested the production of chemokines by lung epithelial cells treated with *S. aureus* in 2D and 3D culture conditions. IL-8 secretion by MLE-12 cells was increased in cells cultured with *S. aureus* in both 2D and 3D culture conditions (Figure 7C). MLE-12 cells treated with *S. aureus* in the PCL-NM, but not on culture dishes, stimulated the secretion of tumor necrosis factor (TNF)-α and IL-1α (Figure 7D). This result indicated that *S. aureus* infection of epithelial cells in 3D culture condition induces proinflammatory response.

## 4. Discussion

The ability of phagocytes to uptake bacteria in vitro may depend on culture conditions and methodology. To date, no method for evaluating phagocytosis of bacteria in 3D co-culture system, which may mimic bacterial infection in vivo, has been reported. Phagocytosis of *S. aureus* by human neutrophils in suspension has been shown to depend on opsonization, while adherent neutrophils do readily internalize bacteria, independent of opsonization [24]. In addition, persistence of free extracellular bacteria in suspension culture has been shown to the poor killing [24]. Adherence to ECM or plasma proteins of host cells is mediated by bacterial surface proteins. In addition, fibronectin-binding proteins of *S. aureus* bind to fibronectin on the surface of epithelial cells [29]. Bacterial adhesion to nanofibers occurs by a number of mechanisms. The adhesion of bacteria to nanofibers is influenced by the surface chemistry, fiber morphology, fiber diameter and physical characteristics of the fiber surface [30]. In addition, specific interactions between bacteria and nanofibers involve macromolecules on the bacterial surface, such as fimbriae and pili [31]. Nanofibers with an average diameter close to bacterial sizes were found to offer support for bacterial adhesion [31], although it is unknown which protein mediates the binding. In this study, *S. aureus* and phagocytes adhered onto the surface of electrospun PCL nanofibers and infiltrated the PCL-NM, allowing 3D evaluation of phagocytosis in PCL-NM-based in vitro culture system. The cell number and ratio of phagocytes to bacteria were the same in 2D and 3D culture conditions, but phagocytosis was lower in 3D than in 2D conditions. In addition, the effect of FBS on the phagocytosis of *S. aureus* by neutrophils in the PCL-NM was more evident than that in culture dishes. Lu et al. demonstrated that more *S. aureus* are phagocytosed by adherent neutrophils compared with those in suspension and that serum opsonization of *S. aureus* enhances phagocytosis by neutrophils in suspension, but has no apparent impact on the phagocytosis of bacteria by adherent neutrophils [24]. They suggested that adherent neutrophils are primed for enhanced phagocytosis [24]. Thus, phagocytes adhered to nanofibers may be less primed for phagocytosis than adherent cells on the culture dish. It is also possible that both phagocytes and bacteria adhere to nanofibers and distribute in 3D space, rather than on the 2D surface. Thus, phagocytes need to sense chemotactic signals from bacteria in 3D culture.

Inflammation is characterized by an influx of phagocytes to the site of infection. While neutrophils are very rapidly recruited into infection site, macrophages constitute the majority of immune cells residing in the tissue at steady state. In a mouse infection model, DCs were actively recruited to infected tissue and phagocytosed *S. aureus*, but they did not actively kill the bacteria [32]. Phagocytes reach the site of infection through directed migration along an increasing gradient of chemoattractants, which are derived from bacteria as well as secreted by activated cells including leukocytes and epithelial cells. In vitro studies have been crucial in understanding cell migration, but the direct exposure of phagocytes to bacteria is in most cases poorly justified. Methods such as Transwell and microfluidic-based assays have been used to evaluate the migration of immune cells in response to chemokines and the migration of phagocytes in vitro is usually measured by chemotaxis through a filter in Transwell chambers. Neutrophils transmigrate through the pulmonary alveolar-endothelial barrier by passing through pores in the PDMS membrane and engulf bacteria in a lung-on-a-chip microfluidic system [9]. When compared to the Transwell assay and lung-on-a-chip microfluidic system, the 3D PCL-NM system has some advantages. First, it is easy to observe the movement of phagocytes over time in the PCL-NM. Second, 3D migratory behavior of neutrophils, macrophages and DCs can be evaluated. We found that neutrophils and DCs on nanofibers become elongated and motile in the vicinity of bacteria and travel along the nanofiber to reach the nearest bacteria. Finally, it is easy to quantitatively evaluate the rates of migration of phagocytes and the phagocytosis of bacteria.

Cell components released from *S. aureus* may act as a phagocyte chemoattractant and bacteria-activated phagocytes secrete cytokines and chemokines, thereby sustaining and prolonging inflammation [33,34]. It has been demonstrated that FPR is required for neutrophil migration to and killing of bacteria, but not for bacterial phagocytosis [35]. This study showed that FPR inhibitors partially inhibited phagocytosis of *S. aureus* by neutrophils and macrophages in 3D, but not in 2D culture, suggesting that FPR agonist released from bacteria may be involved in the detection of bacteria by phagocytes only in 3D culture. Host-derived chemotactic factors, such as IL-8 have been shown to recruit additional neutrophils to areas of infection [36]. This chemokine may be crucial during the early phase of bacterial infection. Thus, the first wave of neutrophil migration is dependent on bacterial chemokines and the second wave of migration of neutrophils, macrophages and DCs is dependent on chemokines secreted from already migrated neutrophils in the infected regions. In this study, the migration of phagocytes through the microstructure of the PCL-NM should be correlated to chemotactic gradients. In this aspect, there was increased secretion of chemokines in the PCL-NM, indicating that phagocytes were further recruited to chemokine-secreting cells via a chemoattractant gradient in the PCL-NM-based two-layer system. Therefore, the compartmentalization and directional orientation of the two-layer system can be applied for the analysis of migration of phagocytes in response to a chemotactic gradient.

Neutrophils and macrophages migrated to *S. aureus*-infected lung epithelial cells in the PCL-NM-based two-layer culture system. By using this system, we can compare the capacity of various bacteria in addition to *S. aureus* to cause phagocyte influx into an infected area. The secretion of TNF-α and IL-1α was increased in lung epithelial cells cultured with *S. aureus* in 3D, but not in 2D culture. TNF-α functions as an initiator of the inflammatory response. Thus, an in vivo inflammation-mimicking response occurred in 3D culture condition. In addition to neutrophil migration to the lower PCL-NM containing epithelial cells and bacteria, epithelial cells were also detected in the upper PCL-NM. The detachment of *S. aureus*-infected epithelial cells was observed only in the presence of migrating neutrophils from the upper to the lower PCL-NM. It is possible that activated neutrophils produce reactive oxygen species and release cytoplasmic granules, which results in significant host cell damage or death as well as the killing of bacteria. This finding suggests that epithelial integrity is lost in 3D culture condition, as observed in infected lung tissue.

In conclusion, the PCL-NM-based culture system provides a 3D space for cell attachment and migration. In the PCL-NM, cell-derived products can diffuse easily and tight cell-to-cell contact can be avoided. Technically, the PCL-NM can be easily assembled into the two-layer system, which mimics multilayers of cells in tissue. Moreover, PCL nanofibers are inert and biocompatible in immune cell culture because the adhesion of DCs to PCL nanofibers does not affect their activation status [12,13]. Finally, 3D migration to surrounding bacteria and phagocytosis processes in the PCL-NM can be visualized though a live cell-imaging setup. Thus, our PCL-NM-based 3D culture system is widely applicable to the biomimetic study of various microbe infections.

## Data Availability

Not Applicable.

## Supplementary Materials

The following are available online at https://www.mdpi.com/article/10.3390/membranes11080569/s1, Figure S1: Workflow for setup of a migration assay with two layers of PCL-NMs. Video S1: Time-lapse images of neutrophils cultured with *S. aureus* on a culture dish depicting phagocytosis of PKH67-labeled *S. aureus* (green) by CellTracker red-labeled neutrophils (red). Video S2: Time-lapse images of DCs cultured with *S. aureus* on a culture dish depicting phagocytosis of PKH67-labeled *S. aureus* (green) by CellTracker red-labeled DCs (red). Video S3: Time-lapse images of neutrophils cultured with *S. aureus* in PCL-NM depicting phagocytosis of PKH67-labeled *S. aureus* (green) by CellTracker red-labeled neutrophils (red). Video S4: Time-lapse images of DCs cultured with *S. aureus* in PCL-NM depicting phagocytosis of PKH67-labeled *S. aureus* (green) by CellTracker red-labeled DCs (red).
