# Supplementary figures and images for: Bacterial Infection-Mimicking Three-Dimensional Phagocytosis and Chemotaxis in Electrospun Poly(ε-caprolactone) Nanofibrous Membrane
Source: Membranes (Basel). 2021 Jul 28;11(8):569. doi: 10.3390/membranes11080569 (PMC8399938; doi:10.3390/membranes11080569)

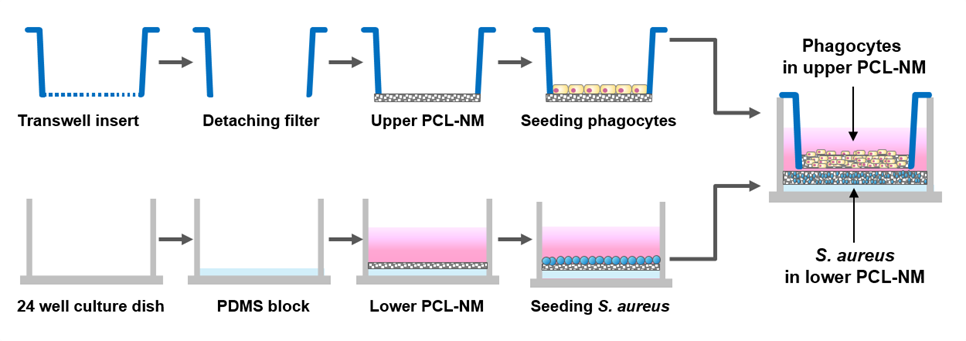

Supplement: Supplementary file 1 [file membranes-11-00569-s001.zip › Membranes-1299691 supplementary/Figure S1. Workflow for setup of a migration assay with two layers of PCL-NMs..tif]
